# Biotechnology in Agro-Industry: Valorization of Agricultural Wastes, By-Products and Sustainable Practices

**DOI:** 10.3390/microorganisms13081789

**Published:** 2025-07-31

**Authors:** Sandra de Oliveira Silva, Amanda Kelly Cristiano Mafra, Franciele Maria Pelissari, Leandro Rodrigues de Lemos, Gustavo Molina

**Affiliations:** 1 Laboratory of Green Materials, Institute of Science and Technology, Universidade Federal dos Vales do Jequitinhonha e Mucuri (UFVJM), Diamantina 39100-000, Minas Gerais, Brazil; 2 Laboratory of Food Biotechnology, Institute of Science and Technology, Universidade Federal dos Vales do Jequitinhonha e Mucuri (UFVJM), Diamantina 39100-000, Minas Gerais, Brazil; 3 Laboratory of Chemical Separations, Department of Chemistry, Universidade Federal dos Vales do Jequitinhonha e Mucuri (UFVJM), Diamantina 39100-000, Minas Gerais, Brazil

**Keywords:** agricultural residues, biotechnological valorization, circular economy, sustainable innovation, industrial by-products

## Abstract

Agricultural and industrial residues are increasingly recognized as valuable resources for sustainable innovation, offering significant potential for biotechnological applications. By integrating waste valorization into production systems, this approach aims to mitigate environmental impacts and enhance economic value across various sectors. The findings underline the critical need for further research and policy support to scale these solutions, advancing global sustainability goals through innovative resource management. In this perspective, this article reviews the utilization of key by-products, including coffee residues, sugarcane bagasse, whey, cassava wastewater (manipueira), and brewery waste, highlighting their transformation into high-value products such as biofuels, bioplastics, enzymes, bioactive compounds, and organic fertilizers. The discussion presented encompasses the challenges and opportunities in leveraging these residues, emphasizing the role of advanced technologies, intellectual property, and circular economy principles.

## 1. Introduction

Agro-industry encompasses all activities involved in transforming raw materials derived from agriculture, livestock, aquaculture, and forestry, with varying degrees of processing depending on the objectives and the products intended for consumers [1]. According to the Brazilian Confederation of Agriculture and Livestock (CNA), Brazilian agricultural production has grown substantially, positioning the country as a future major food supplier. In 2022, agribusiness accounted for 25% of the national GDP, totaling BRL 2.54 trillion, of which 72.2% (BRL 1.836 trillion) originated from agriculture and 27.8% (BRL 705.36 billion) from livestock [2]. This growth also results in a significant impact on waste generation.

The transformation of agro-industrial products generates tons of organic waste along the production chain, causing environmental impacts due to accumulation and improper disposal [3]. The Food and Agriculture Organization (FAO) estimates that global agro-industrial waste production reaches 1.3 billion tons annually [4]. Due to the large volume, difficult transport, low added value, and microbial growth, such waste is primarily used as animal feed, fuel, or disposed of in landfills [5].

The definition of waste and by-products is controversial. Industrial waste derives from industrial processes and differs from domestic waste [6]. Solid waste includes process leftovers, disposals, and packaging trash, categorized as organic—originating from agricultural and livestock activities (crop residues, animal waste, and agro-industrial effluents)—or inorganic, such as pesticide and fertilizer packaging and rural household waste [4]. By-products, on the other hand, are substances resulting from production processes that can be commercialized without environmental or health impacts, adding value to what was previously considered waste [6].

The continuous search for alternatives to use agro-industrial waste by transforming it into high-value-added by-products is a constant priority across all production chains, incorporating sustainability into their processes [7]. In this context, biotechnology plays a crucial role in reducing agro-industrial waste, offering innovative and sustainable solutions.

To address this challenge, the bioconversion of agro-industrial waste into commercial products, through processes such as fermentation, proves effective. These wastes, abundant and low-cost, are ideal for biorefineries, addressing the need for waste disposal sites while transforming problems into profit [8]. Due to their composition being rich in fermentable sugars and nutrients, these wastes serve as ideal substrates for microorganisms, converting them into high-value-added products [9].

Fermentation can occur in two forms: solid-state fermentation, where the substrate is fermented under moistened solid conditions with a thin liquid film, and submerged liquid fermentation, where microorganisms are cultured in nutrient-rich liquid media within closed and agitated reactors. Parameters such as temperature, pH, oxygen consumption, and carbon dioxide formation are monitored for optimization [10].

The conversion of agro-industrial wastes into high-value products is grounded in biotechnological advancements that enable the transformation of nutrient-rich materials and bioactive compounds into inputs for the food, pharmaceutical, cosmetic, and energy industries [11,12,13,14]. These traditionally discarded residues represent an abundant and low-cost source for the production of biofuels, enzymes, biopolymers, antioxidants, vitamins, single-cell proteins, and other compounds of industrial interest [11,13,14,15]. The use of processes such as solid-state fermentation and innovative extraction and bioconversion technologies enhances the recovery of high-value molecules, promoting sustainability and reducing environmental impacts [12,14,15]. Furthermore, the valorization of these residues contributes to the circular economy, creating new business opportunities, generating employment, and adding value to production chains [11,16,17]. The integration of agro-industrial residues into microbial bioprocesses also drives bioeconomy policies and can be a catalyst for regional development, particularly in agricultural-producing countries [14,16].

By transforming by-products and waste into biofuels, fertilizers, and animal feed, biotechnology not only reduces environmental impact but also promotes economic efficiency, even protecting innovations through intellectual property. This legal field encompasses copyrights, trademarks, and patents, which ensure creativity and innovation across various areas. Patents, specifically, grant inventors’ temporary monopolies, allowing them to commercially exploit their creations while publicly disclosing the details, advancing knowledge and technology [18,19].

Studies indicate that applying biotechnological processes in agro-industries can convert waste into valuable resources, promoting sustainability and economic feasibility in the sector. Therefore, this study aims to conduct a bibliographic review on the valorization of waste through biotechnology, considering process conditions and the products obtained.

Table 1 below provides a synthesis of the main residues discussed throughout the text, highlighting the products generated, the most commonly applied microorganisms, and the biotechnological processes involved in their valorization.

## 2. Cassava (*Manihot esculenta* Crantz): Production, Industrial Applications, and Waste Management

Cassava (*Manihot esculenta* Crantz), also known as manioc, yuca, or macaxeira, is a native plant of South America and widely cultivated in various tropical countries. Belonging to the Euphorbiaceae family, cassava is a staple food for over 700 million people worldwide [20,21]. Originating in Brazil, cassava is one of the richest sources of starch globally [22]. Approximately 100 countries produce cassava, with Brazil ranking as the fourth-largest global producer, yielding about 17 million tons annually, with a harvested area of roughly 1 million hectares [23]. According to the Systematic Survey of Agricultural Production (LSPA) and the Brazilian Institute of Geography and Statistics (IBGE), production in 2023 reached 18.67 million tons over a total area of 1.28 million hectares, with production concentrated in Pará (22%) in northern Brazil and Paraná (17.62%) in southern Brazil [24].

Cassava is primarily cultivated by small-scale farmers and serves various purposes, including flour, starch, and direct consumption. Its cultivation is more prominent in tropical regions due to its adaptability to diverse climatic conditions and soil types [25]. In addition to being consumed directly as food, cassava roots are processed into basic products such as table flour and starch (commonly known as sweet starch or tapioca flour). This starch finds applications in multiple industries, including food, pharmaceuticals, paper, and chemicals, often undergoing different modification processes [21]. In 2022, Brazil exported 43.6 thousand tons of cassava starch, a 6% increase compared to 2021, marking the second consecutive year of record exports. In June 2023, exports reached 1.5 thousand tons, consistent with the previous two months, with a quarterly average of 1.6 thousand tons—approximately half the monthly average of the first quarter of 2023. Revenue totaled USD 1,709,144, driven by increased volume and prices [24].

Cassava is also utilized in animal feed, either in its raw form or through its by-products, such as peels and starchy residues from processing [26].

In industrial applications, cassava generates various residues composed of putrescible organic matter. When improperly disposed of, these residues can negatively impact natural resources and pose risks to public health [27,28]. The primary residues include solids, which constitute 18% of the root, such as peels, inner peels, crueira (coarse meal), fiber, and bagasse, and liquid residues, which account for 60–70% of the root, such as washing water (manipueira) and starch extraction water [27,29].

**Figure 1:**
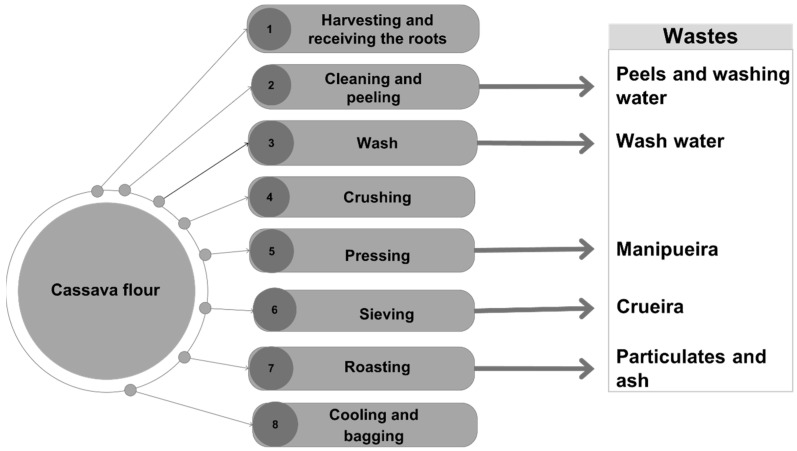
Cassava waste generation during the processing for flour production (adapted from [31]).

According to Andrés-Meza et al. [30], producing one ton of starch generates approximately 2.5 tons of residues, with peel production ranging between 100 and 300 kg per ton of cassava and about 17.4 m^3^ of wastewater. The stages at which each residue is generated are detailed in Figure 1, which illustrates the cassava processing steps for flour production.

Table 2 provides a detailed composition of cassava processing residues (in g/100 g of dry matter) and their mineral content (in mg/kg of dry matter) across different types of residues: bagasse, cassava wastewater (manipueira), peels, and effluent. These data are essential to understanding the nutritional and mineral composition of these residues and determining their best applications.

The bagasse shows a high crude fiber content (19.3 g/100 g) and moisture (84.2 g/100 g), along with being rich in potassium (138 mg/kg) and magnesium (129 mg/kg). Cassava wastewater is notable for its elevated moisture content (96.7 g/100 g) and a significant amount of potassium (42.7 mg/kg). The peels contain the highest concentration of crude fiber (29.6 g/100 g) and are particularly rich in potassium (269 mg/kg) and calcium (122 mg/kg). The effluent, a mixture of serum and fibers, has a significant moisture content (91.4 g/100 g) and contains minerals such as iron (16.2 mg/kg) and sodium (24 mg/kg).

The analysis of Table 2 clearly highlights the significant potential of cassava residues, attributed to their high nutrient content and organic matter. These resources, characterized by being affordable and low-cost raw materials, can be applied in various high-value-added areas. In this context, biotechnology emerges as a key ally, capable of mitigating the negative environmental impacts associated with these residues by enabling their use in agro-industry for the production of higher-value-added products [30,33]. Thus, these residues are utilized as carbon sources for microbial growth, primarily during fermentative processes, resulting in the production of metabolites relevant to biotechnology [34]. Below, some applications of these residues found in the literature are discussed.

Cassava wastewater plays a crucial role in the production of biosurfactants. Rich in carbon and essential nutrients, cassava residues serve as raw materials for the synthesis of various biosurfactants [35]. Biosurfactants are compounds with surface-active properties that reduce the surface tension between liquid–gas phases or the interfacial tension between immiscible liquids [36]. The study by Schmidt et al. [35] investigates the production of biosurfactants such as surfactin (a lipopeptide), rhamnolipids (glycolipids), and mannosylerythritol lipids, using cassava wastewater as a culture medium. For example, *Bacillus subtilis* is employed in surfactin production, while *Pseudomonas aeruginosa* synthesizes rhamnolipids. Additionally, various microorganisms, such as *Schizonella melanogramma* and *Pseudozyma* sp., can be used to produce mannosylerythritol lipids. During controlled fermentation, these microorganisms metabolize the nutrients present in the wastewater, generating biosurfactants as secondary metabolites, which are subsequently extracted and purified. Table 3 provides an overview of the biosurfactants produced using cassava wastewater as a nutrient source, as well as the process conditions, mode of operation, and yields.

The data show that different cultivation conditions and operational modes influence biosurfactant yields. For example, the lipopeptide produced by *Bacillus subtilis* LB5a in an Erlenmeyer flask yielded 3.0 g/L under conditions of 30 °C and 150 rpm for 72 h.

In a bioreactor, under conditions of 35 °C, 150 rpm for 60 h, and an aeration rate of 0.63 vvm, the yield was 2.40 g/L. In turn, the glycolipid (MEL-B) produced by *Pseudozyma tsukubaensis* in a bioreactor yielded 1.26 g/L, with variations in agitation and aeration conditions over time.

Maia et al. [39] investigated the use of cassava wastewater and frying oil residues as substrates for *Bacillus subtilis* UCP 099 to produce biosurfactants. Using a full factorial design, the study achieved a minimum surface tension of 33.2 mN/m and a maximum emulsification index of 95%, using 5% cassava wastewater and 2% residual frying oil. The process conditions included a 96 h incubation under orbital agitation at 150 rpm and 30 °C, resulting in a yield of 2.67 g/L. The biosurfactant was identified as anionic and polypeptidic. On the other hand, Bezerra [40] used a fractional factorial design (2(4-1)), utilizing cassava wastewater as a carbon source with *Pseudomonas aeruginosa* AP029—GVIIA in batch submerged cultivation. Carbon consumption ranged from 55% to 90%, with optimal conditions achieved at 200 rpm, 30 °C, and 0.4 aeration. This setup resulted in significant surface tension reduction (30.08%) and a minimum surface tension of 30.98 mN/m. Additional studies on biosurfactant production using these residues are found in Makkar et al. [41].

Studies by Martins et al. [42], Cruz et al. [43] and Kehinde et al. [44] emphasize the relevance of cassava residues in biogas production, primarily methane (CH_4_). This transformation occurs in anaerobic digesters, where microorganisms play a fundamental role in degrading organic matter. Anaerobic digestion comprises four stages: hydrolysis, acidogenesis, acetogenesis, and methanogenesis. Hydrolytic bacteria such as *Clostridium* and *Bacteroides* break down complex structures into simple components. Acidogenic bacteria like *Lactobacillus* and *Streptococcus* ferment these products into organic acids, alcohols, and CO_2_. Acetogenic bacteria, such as *Syntrophomonas* and *Syntrophobacter*, convert these acids and alcohols into acetic acid, hydrogen, and CO_2_. Methanogenic archaea, such as *Methanobacterium*, *Methanosaeta*, and *Methanosarcina*, then produce methane and water. The methane generated can be captured and used as renewable energy, while the effluent serves as a nutrient-rich biofertilizer [43,44].

In a specific study using a batch-fed digester, cassava peels were employed for biogas production. A total of 60 kg of peels and ¾ water filled the digester tank for 40 days, with periodic manual agitation to maintain adequate contact between microorganisms and substrates. After 40 days, 1.94 dm3/60 kg of residues was obtained, with an average yield of 0.048 dm^3^/day. Methane presence was confirmed by the blue flame, with temperatures ranging from 27 to 33 °C and a pH of approximately 3.21 [44].

In another study by Ismail et al. [45], cassava peels and stems were used in an anaerobic digester with a 2 L working volume, employing co-digestion with naturally composted seeds. Cassava peel fermentation yielded higher biogas (approximately 1000 mL) than stems, attributed to higher total suspended solids, a biochemical oxygen demand of 0.28 mg/L, and a chemical oxygen demand of 378 mg/L.

The production of bioethanol from cassava residues has also been extensively studied [46,47]. Enzymatic hydrolysis converts cassava bagasse into fermentable sugars, serving as a carbon source for ethanol production by *Saccharomyces cerevisiae* [48] reported a yield of 0.25 m^3^ ethanol per ton of bagasse.

Biotechnology not only mitigates environmental impacts but also fosters sustainability and circularity in agro-industry. Transforming cassava residues into high-value products like biosurfactants, biogas, bioethanol, and biofertilizers creates new economic and technological opportunities for the sector, advancing innovation and environmental stewardship.

### Technological Advances Through Intellectual Property Analysis

Intellectual property is a field encompassing copyrights, trademarks, and patents. These rights play a crucial role in fostering creativity and innovation across various domains, ranging from literature and entertainment to technology and industry. Specifically, patents hold a significant role in protecting intellectual property. Patents grant inventors a temporary monopoly over their inventions, allowing them exclusive commercial exploitation for a specified period. In return, inventors publicly disclose the details of their inventions, contributing to the advancement of knowledge and technology [18,19].

To identify relevant biotechnological innovations in the valorization of agricultural residues, a systematic patent search was conducted using the ESPACENET database and Google Patents. Specific keywords and International Patent Classification (IPC) codes related to the biotransformation of agro-industrial waste, such as biofuels, enzymes, organic fertilizers, and others, were employed to refine the search. The scope of patents is diverse, covering industrial processes and innovative products. In the specific context of cassava residues, some patent applications have already been registered. Table 4 highlights a sample of relevant patents selected to illustrate current trends in this domain.

The data demonstrates significant interest in exploring cassava residues for various industrial applications. These patents highlight the potential of cassava residues as valuable resources for technological innovation and industrial sustainability. The utilization of these residues fosters a circular economy, where by-products are repurposed in new production processes. This represents a significant advancement in the pursuit of more eco-friendly and efficient solutions, aligning with global trends in sustainability and innovation.

Despite the potential of cassava residues for the production of biosurfactants, biogas, bioethanol, and other biotechnological products, several challenges still hinder their implementation at an industrial scale. The economic viability of these routes depends on the regular supply and efficient logistics for waste collection, which are often scattered across small rural properties, increasing costs and making continuous supply to industrial plants difficult [53,54,55]. Additionally, high infrastructure costs for fermentation, purification, and environmental control present significant barriers to scaling the processes, especially in regions with limited access to advanced technologies [35,53,55]. The presence of toxic compounds, such as cyanide in manihot wastewater, requires specific and stringent treatments to meet environmental standards and avoid public health risks, which can further increase operational costs [35,53,56]. Environmental regulation, therefore, imposes additional restrictions, requiring proper monitoring and management of the residues [53,57]. Another hurdle is the low integration between research centers, farmers, and the industrial sector, which hinders technology transfer and the adoption of large-scale innovative solutions [54,55].

## 3. Valorization of Orange Residues: Biotechnological Applications and Potential

Half of the total food waste comes from fruits, vegetables, and roots, which are rich in carbohydrates, starch, cellulose, soluble sugars, minerals, and organic acids [58]. These characteristics make them a favorable medium for biotechnological applications and the generation of high-value-added products. Non-edible parts of fruits, such as peels and seeds, are classified as agro-residues and are often discarded in the environment. However, these residues may contain higher nutritional value than the edible portions of the fruit [6]. Among fruits with significant industrial applications, oranges stand out.

The orange (*Citrus sinensis*) is a natural hybrid resulting from the cross between pomelo (*Citrus maxima*) and mandarin (*Citrus reticulata*). It is cultivated in tropical and subtropical regions worldwide and possesses nutraceutical properties, such as vitamin C, which strengthens the immune system and benefits bones, cartilage, muscles, and blood vessels, contributing to the prevention of chronic diseases [59]. In Brazil, the production value of oranges in 2022 reached BRL 14.37 billion, with a total production of 16.93 million tons and a harvested area of 568,132 hectares [60]. Brazil is the world’s largest producer of oranges and orange juice, accounting for 33.9% of global orange production and 67.8% of the global juice volume, followed by China and the European Union [61]. São Paulo and Minas Gerais, home to the country’s largest citrus production hub, account for approximately 70% of the cultivated area and 83% of national orange production [61]. During the orange processing industry, the primary residues generated are peels, pulp, and seeds [62], amounting to 8.8 million tons of waste annually in Brazil [63]. These residues, being rich in biopolymers and bioactive compounds such as proteins, carbohydrates, lipids, lignin, polyphenols, and natural pigments, have the potential to be converted into high-value chemicals, including bioplastics, functional materials, and biofuels [63]. When analyzed on a dry mass basis, orange peels are composed of 49.59% carbon, 6.95% hydrogen, 0.66% nitrogen, 0.06% sulfur, 3.05% ash, and 2.73% water [64]. Thus, utilizing these residues to produce value-added products becomes a viable and sustainable option.

Xylitol (C5H12O5) is a natural sweetener found in certain foods and commonly added to food products. It belongs to the sugar alcohol or polyol carbohydrate class, with sweetness comparable to sucrose (common sugar) [65]. Xylitol is obtained from fibers present in various plants, and its production process involves the catalytic hydrogenation of xylose. This compound is widely used in candies, confectioneries, chewing gums, and other similar products [66]. Among the sugars present in orange residues, D-xylose is notable. Like other monosaccharides, xylose must be transported from the extracellular environment into yeast cells to enable fermentation. Once inside the cell, this pentose sugar is reduced to xylitol, which is subsequently oxidized to xylulose [47]. In addition, xylooligosaccharides (XOs) are sugar oligomers composed of xylose units that can be obtained through acid or enzymatic hydrolysis [67]. In the study by Gupta et al. [67], orange peels were used as a substrate for the cultivation of *Aspergillus niger* (MTCC 281) to produce xylanase. The peels served as the sole carbon source, using 1 g/10 mL of peels autoclaved at 121 °C for 15 min. The A. niger was inoculated and cultivated in a rotary incubator for 5 days at 40 °C, achieving a yield of 558 mg/mL of XOs in the liquid medium.

Citric acid, an organic acid, can be obtained from sources such as lemon or pineapple juice or through the fermentation of carbohydrate solutions or other suitable media using Candida spp. or non-toxigenic strains of *Aspergillus niger* [68]. This acid is found in a variety of acidic fruit juices, including orange juice. In this context, the study by Torrado et al. [69] explored the use of Valencia orange (*Citrus sinensis*) peel as a raw material for citric acid (CA) production through solid-state fermentation (SSF) with *Aspergillus niger* CECT-2090 (ATCC 9142, NRRL 599) in Erlenmeyer flasks. The optimal conditions for CA production were determined to be an inoculum of 0.5 × 10^6^ spores per gram of dried orange peel, a bed load of 1.0 g of dried orange peel, and an initial humidification level of 70% of the maximum water retention capacity (MWRC), followed by the addition of 0.12 mL of H_2_O per gram of dried orange peel (equivalent to 3.3% of the MWRC) every 12 h after 62 h. Under these conditions, SSF achieved an effective specific production of 193 mg CA per gram of dried orange peel. In the study conducted by Kuforiji et al. [70], orange and pineapple pulp residues were used as substrates for citric acid production employing two strains of *Aspergillus niger* (NRRL 567 and NRRL 328). Using *A. niger* NRRL 567, yields were 57.6% with orange residues and 46.4% with pineapple residues, whereas *A. niger* NRRL 328 achieved yields of 55.4% with orange residues and 45.4% with pineapple residues. Similarly, Hamdy [71] investigated citric acid production using a culture medium containing orange peels fortified with cane molasses to increase sugar concentration in the system, promoting fermentation with *Aspergillus niger*. Maximum citric acid yield reached 640 g per kilogram of orange peel under conditions of 72 h of incubation with peels hydrated to 65% *w*/*v*, a bed load of 20%, an initial pH of 5, a temperature of 30 °C, an agitation rate of 250 rpm, and molasses fortification to achieve a final sugar concentration of 14%, with the addition of 3.5% methanol.

Another ingredient with significant potential for the food industry is xanthan gum, a high-molecular-weight anionic polysaccharide produced industrially by the bacterium *Xanthomonas* sp. This gum forms pseudoplastic solutions, exhibiting favorable flow properties and contributing to excellent formulation stability [72]. In a study conducted by Mohsin et al. [73], a response surface methodology and kinetic modeling approach were employed to optimize xanthan gum production. In this process, hydrolyzed orange peel was used as a carbon source. The optimized parameters included 1.62% acid hydrolysis, 85% carbon source derived from hydrolyzed orange peel, and a temperature of 30.4 °C. The optimized treatment was performed in a batch culture fermenter. Notably, xanthan gum production in the bioreactor reached 30.19 g/L, with conversion rates of 69.29% and reducing sugar utilization of 99.99%.

In a study conducted by Ricci et al. [74], the efficiency of orange peels in lactic acid production was investigated. Using the solid-state fermentation (SSF) technique, 5 g of dried solid substrate composed of orange peels were utilized. After autoclaving (20 min at 120 °C and 1.2 atm), the pH was adjusted to either 5 or 6.5. The initial microbial culture was inoculated onto the orange peel substrate at a concentration of approximately 7 log colony-forming units per gram. SSF was carried out over five days, maintaining the optimal growth temperature for the microorganisms: 37 °C for *Lactobacillus rhamnosus*, *Lactobacillus casei*, and *Lactobacillus paracasei*, and 30 °C for *Lactobacillus plantarum* and co-cultures. The most efficient strain in terms of yield was *Lactobacillus casei* 2246, which achieved the highest lactic acid concentration (209.65 g kg^−1^) and yield (0.88 g g^−1^).

In a study conducted by Bustamante et al. [75], strain cultivation was performed in 50 mL tubes containing MRS medium with 85% *v*/*v* hydrolyzed orange peel. Incubation temperatures were set to 37 °C and 45 °C under microaerobic conditions. All experiments began with 15% *v*/*v* pre-culture inoculation and were incubated in an orbital shaker at 200 rpm for 120 h. Bioreactor experiments were conducted in a 1.5 L batch system. Hydrolyzed orange peel (85% *v*/*v*) was used as the carbon source, with 5 g/L of meat extract added to the MRS medium as an additional nitrogen source. Before inoculation, an anaerobic atmosphere was created by removing oxygen with a nitrogen flow. Experimental conditions were set at 37 °C, 200 rpm, and pH 5.8, with adjustments made using 5 M NaOH or 2 M HCl to maintain pH during fermentation. Notably, *Lactobacillus* delbrueckii ssp. bulgaricus CECT 5037 exhibited the best performance, achieving a yield of 84% *w*/*w*. Other studies have also explored lactic acid production using orange residues, including those by Fazzino et al. [76] and Torre et al. [77].

In addition to the products already mentioned, other valuable compounds can be extracted from orange peel residues, as shown in Table 5.

Table 5 highlights the diversity of products that can be extracted from orange peel using various extraction methods. Pectin, for instance, can be extracted using microwave-assisted methods or conventional acid techniques, yielding 485 mg/mL and 381 mg/mL, respectively. Ultrasonication with ethanol as the extraction solvent is highly effective for obtaining flavonoids, resulting in 205.2 mg of hesperidin per 100 g of fresh weight. Carotenoids are extracted using ultrasound and d-limonene, achieving a yield of 11.25 mg/L. Finally, microwave-assisted steam distillation is employed to obtain essential oil.

### Technological Advances Through Intellectual Property Analysis

Significant advancements have been observed in the development of intellectual property related to the technological utilization of oranges and their by-products. These innovations highlight the growing interest in leveraging the full potential of citrus residues, aligning with global trends toward sustainability and value-added processing. The expansion of patents in this field demonstrates the increasing importance of circular economy principles and also underscores the role of technology in transforming waste into high-value products. Table 6 presents patents related to orange peel residues, demonstrating significant technological advancements in the utilization of these residues.

Table 6 highlights technological innovation in the utilization of orange peel residues. The listed patents cover a wide range of applications, including the production of immune-enhancing feed additives and xylooligosaccharides, the preparation of cellulose fibers, and the synthesis of ethene from citrus waste. These advancements not only demonstrate the economic potential of orange peel residues but also contribute to sustainability by promoting the comprehensive use of natural resources.

Although orange residues have significant potential for the production of high-value compounds, their application on an industrial scale faces considerable challenges. Economic viability depends on the standardization and continuous supply of these residues, which are hindered by the seasonality of production and the fragmentation of the agro-industrial chain, compromising supply regularity [83,84]. Furthermore, the biotechnological processes required for the extraction and conversion of these compounds are complex, necessitating specialized equipment, controlled conditions, and additional inputs, thereby increasing production costs [85,86]. The variability in the composition of the residues and the lack of standardized processing methods also complicate the consistent production of high-quality products [83,84,87]. The final products must meet stringent quality and safety standards, especially for food and pharmaceutical use, which implies additional regulatory challenges [86,88]. The absence of specific regulatory frameworks for waste-derived products can delay market entry and increase uncertainty for investors and industries [83,86].

## 4. Biotechnology Applied to Sugarcane Bagasse for the Production of Economically Valuable Products

Sugarcane (*Saccharum officinarum*) is a plant of great economic and environmental importance, ranking among the most widely cultivated agricultural crops globally, particularly in tropical and subtropical regions. Native to Asia, sugarcane is extensively used for producing sugar, ethanol, and other high-value by-products, while also playing a significant role in renewable energy and biotechnology industries. Belonging to the family *Poaceae*, *Saccharum officinarum* is primarily grown in countries such as Brazil, India, and China, where the climate is ideal for its cultivation [89]. Sugarcane originated in New Guinea and was gradually disseminated to other parts of Asia before being introduced to the Western world during the colonial expansion [90].

The primary use of sugarcane is sugar production, accounting for approximately 80% of the world’s sugar supply [91]. In Brazil, the largest sugarcane producer globally, the sugar–ethanol industry forms one of the pillars of the national economy, generating millions of direct and indirect jobs. Sugarcane is rich in sucrose, which is extracted from its juice to produce white and brown sugar. After extraction, the bagasse is utilized as a biomass source for energy cogeneration in mills [92]. Another widely recognized application of sugarcane is ethanol production, a biofuel that has become a sustainable alternative to fossil fuels. Ethanol is produced through the fermentation of sugars present in sugarcane juice by yeast, a process that converts sucrose into alcohol. Sugarcane ethanol is renowned for its high energy efficiency and lower greenhouse gas emissions compared to petroleum-derived fuels [93]. In Brazil, ethanol has been established as an alternative to gasoline, driven by the Proálcool program, launched in the 1970s [91].

Sugarcane bagasse, the fibrous residue left after juice extraction, has become a significant source of renewable energy. It is utilized in cogeneration processes at mills, where it is burned in boilers to produce steam, which subsequently drives turbines to generate electricity [89]. This process not only helps reduce reliance on non-renewable energy sources but also generates surplus electricity that can be sold to the national power grid, contributing to the sustainability of the energy matrix. In addition to sugar and ethanol, sugarcane and its by-products have demonstrated considerable potential in various biotechnological applications. For instance, bagasse can serve as a raw material to produce bioplastics and renewable chemicals, such as lactic acid, which is used in the manufacturing of biodegradable plastics [94]. Sugarcane straw is also being investigated for the development of bioactive compounds, such as antioxidants and dietary fibers, which have applications in the food and pharmaceutical industries [95,96].

The sugarcane industry is one of the most important sectors of the Brazilian economy, with Brazil being the largest global producer of sugarcane. In 2023, the country produced approximately 715 million tons of sugarcane, representing around 40% of global production [97]. Beyond being a critical source of sugar, sugarcane is utilized for ethanol production, a key component of Brazil’s energy matrix. The sector significantly contributes to the country’s agricultural GDP and employs hundreds of thousands of workers, primarily in the Southeast and Midwest regions [98].

**Figure 2:**
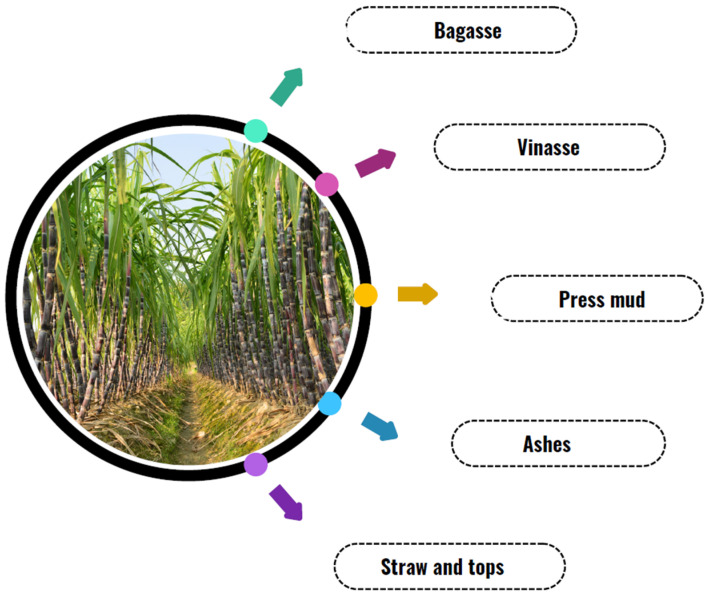
Main residues of sugarcane production—adapted from [96,98,99].

According to Sahu [99], sugarcane production generates a substantial amount of waste at various stages of the production process, as illustrated in Figure 2.

These residues are generated at various stages of the process, ranging from harvest (leaves and tops) to industrial processing (bagasse, vinasse, filter cake, and ash). Proper management of these residues is essential to minimize environmental impact and promote the sustainability of the sector [96,98,99].

Sugarcane bagasse is the primary fibrous residue obtained after pressing the stalks to extract juice. Processing one ton of sugarcane yields approximately 0.25 to 0.30 tons of bagasse, with the average in Brazilian mills being 0.28 tons [100]. Globally, it is estimated that more than 700 million tons of bagasse are produced annually, accounting for 40–50% of the total weight of sugarcane processed. Among the top ten producing countries, over 540 million tons of bagasse are generated each year [98].

The chemical composition of sugarcane bagasse varies depending on the plant variety, cultivation conditions, harvesting practices, and processing methods. Typically, it contains 45–50% water, 2–5% dissolved sugars, 40–45% fibers, and 60–80% carbohydrates. Bagasse has a fibrous structure composed primarily of cellulose, hemicellulose, and lignin. Table 7 presents the chemical composition of sugarcane bagasse.

The nutritional composition of sugarcane bagasse is crucial for biotechnological processes, particularly in the production of bioethanol and other biofuels. Bagasse is rich in cellulose, hemicellulose, and lignin, components that can be converted into fermentable sugars following appropriate pretreatment. These sugars are essential for bioethanol production through fermentation [104]. The high concentration of carbohydrates in bagasse makes it an excellent substrate for bioethanol production. However, the recalcitrant nature of bagasse, due to its lignin content, necessitates pretreatment methods such as steam explosion or alkaline treatment to release sugars trapped in the lignocellulosic fibers [105]. Additionally, bagasse can serve as a source for other value-added products, such as enzymes and organic acids, after hydrolysis processes. Leveraging bagasse in this manner is critical not only for improving biofuel production efficiency but also for promoting a circular economy, reducing waste, and sustainably utilizing by-products [106].

Several studies have explored sugarcane bagasse (SCB) as a raw material for producing new products through biotechnological processes. SCB, a by-product of the sugar industry, is not only a renewable source of fermentable sugars but also contains approximately 60% carbohydrates, making it a lignocellulosic biomass with great potential [94]. However, most SCB is still used in energy cogeneration processes. Recent studies suggest that by developing biochemical platforms to produce sugars from SCB, its potential can be unlocked for generating renewable fuels and chemicals, such as lactic acid (LA) [94]. This compound, which has applications in various industries, is considered one of the key platform chemicals by the United States Department of Energy. LA production is primarily dominated by microbial fermentation of starch- or sugar-rich raw materials. Technical advancements in utilizing SCB for this purpose include improvements in pretreatment, saccharification, fermentation, and downstream processing. These advancements are facilitating the transition from laboratory-scale research to industrial-scale production [94].

In another study, Prabhu et al. [107] investigated the production of fibrinolytic enzymes using sugarcane bagasse (SCB) as a low-cost substrate in solid-state fermentation processes. The microorganism *Alcaligenes aquatilis* PJS_1, isolated from slaughterhouse soil samples, was identified as a potential producer of this enzyme. Under optimized conditions, which included glucose as a carbon source and casein as a nitrogen source, the highest production of fibrinolytic enzyme was achieved after 60 h of incubation at 35 °C. This study demonstrated that SCB, combined with glucose and casein, can serve as an efficient medium for enzyme production, with potential medical applications as a thrombolytic agent [107].

Additionally, Tavares et al. [108] explored the genome of the phytopathogenic fungus *Chrysoporthe cubensis*, identifying genes encoding multicopper oxidase enzymes (MCOs), such as laccases. The research demonstrated that the use of these laccases in the saccharification of sugarcane bagasse (SCB) was more efficient than commercial preparations, showcasing their potential for biomass hydrolysis and the detoxification of phenolic compounds [108]. Ridella et al. [109] conducted an innovative investigation into the biocatalytic oxidation of 5-hydroxymethylfurfural (5-HMF) using the strain *Bacillus* nitratireducens, isolated from SCB. The process resulted in the production of 5-hydroxymethyl-2-furancarboxylic acid (HMFCA), a promising compound for polyester production and medical applications. The study achieved a 91% yield, demonstrating the strain’s potential for sustainable and economical utilization of 5-HMF without the need for metallic catalysts [109].

Another example is the study by Bonfiglio et al. [110], which explored the sustainable production of xylitol, a five-carbon sugar alcohol, using the yeast *Wickerhamomyces anomalus* Z1. The yeast underwent an adaptive laboratory evolution (ALE) process, resulting in a strain with higher xylitol production yields compared to the wild strain. This advancement underscores the potential of SCB as a substrate for valorizing hemicellulosic fractions [110]. Similarly, Ferreira et al. [111] evaluated the enzymatic activity of six filamentous fungi strains cultivated with SCB as the sole carbon source, identifying *Aspergillus japonicus* Saito as the most efficient in producing enzymes such as cellulases, xylanases, amylases, and laccases. These enzymes demonstrated significant biotechnological potential for applications across various industries [111].

Barbieri et al. [112] investigated the production of cellulose nanofibrils (CNFs) and glucose from SCB, achieving a reduction in lignin content from 21% to 5% and converting 93% of the cellulose into glucose through enzymatic hydrolysis. This study demonstrated the potential of SCB for nanocellulose production, with applications in various industrial sectors [112]. Finally, Martínez et al. [113] highlighted the importance of reusing lignocellulosic residues in Colombia, where only 10% are currently utilized. Their study focused on the production of hemicellulolytic enzymes through solid-state fermentation of SCB and wheat bran, resulting in a significant yield of xylanases. These enzymes hold promising applications in the silage industry and the saccharification of lignocellulosic materials [113].

The biotechnological valorization of sugarcane residues, especially bagasse, holds significant potential for the production of biofuels, bioplastics, enzymes, and other high-value products, contributing to a circular and low-carbon economy. However, technical and economic challenges persist, such as the high costs of pretreatment processes, the recalcitrance of lignocellulosic biomass, and the need for advanced technologies to enable efficient conversion on an industrial scale [114,115,116]. The competitiveness of these processes compared to traditional inputs is still limited, partly due to the complexity of the bagasse structure, which requires efficient pretreatment methods, often costly or unsustainable [115,116,117]. Moreover, regulatory gaps and the lack of incentive policies hinder the integration of derived products into strategic sectors, such as food and pharmaceuticals [117,118]. Overcoming these barriers requires investments in applied research, the development of integrated biorefineries, and partnerships between academia, industry, and government, fostering innovation and sustainability [115,118,119].

### Technological Advances Through Intellectual Property Analysis

The utilization of sugarcane bagasse plays a pivotal role in developing sustainable solutions across various industries, transforming waste into valuable resources.

As a by-product of sugar and ethanol production, bagasse, once considered waste, is now a strategic resource for energy generation through cogeneration. This process enables sugar and ethanol mills to be energy self-sufficient, with surplus energy exported to the grid. Beyond energy, bagasse serves as a raw material for biotechnological innovations, including the production of second-generation biofuels, biodegradable plastics, and high-value chemicals like lactic acid and xylitol. These advances reduce reliance on fossil fuels, mitigate greenhouse gas emissions, and promote a circular economy by maximizing resource utilization and minimizing waste.

Innovations in this area are often protected through patents, granting exclusive rights for the commercial exploitation of novel products or processes. Typically, valid for 20 years, patents drive industrial advancements by safeguarding inventive steps and industrial applications [120,121]. Table 8 highlights some key patents in this field.

Sugarcane bagasse holds significant potential as a renewable resource for various biotechnological applications, ranging from the production of organic acids and enzymes to advanced materials and chemicals. While advancements in this field are promising, technical and economic challenges must be addressed to ensure the successful transition from laboratory research to industrial-scale production. The comprehensive utilization of sugarcane bagasse can contribute to a more sustainable economy, reducing dependence on fossil resources and promoting the valorization of agro-industrial residues and innovation in biotechnology.

## 5. Valorization of Industrial Residues in Human Nutrition: Toward Sustainability and Innovation

Beer is a popular alcoholic beverage produced through the fermentation of grains, primarily malted barley, by the action of yeast and, in some cases, bacteria. Water is its main component, and the mixture can be enriched by adding other grains, whether malted or unmalted [127]. The origins of beer are believed to be accidental, stemming from the spontaneous fermentation of grains. Over the years, its production process has been refined and transformed, becoming popular in Brazil in the 17th century due to its introduction during Dutch colonization [128]. According to Normative Instruction No. 63 of November 27, 2020, the alcohol content of beer must exceed 2.0% by volume. When produced from a wort, the original extract must contain at least 55% malted barley by weight and a maximum of 45% brewing adjuncts [129].

Brazil is the third-largest beer producer in the world, with a sales volume of 16.1 billion liters [130]. The country has 1847 beer production establishments registered with the Ministry of Agriculture and Livestock (MAPA), reflecting a growth of 6.8%. The highest concentration of these establishments is in the South and Southeast regions, with São Paulo being the leading state, hosting 410 establishments [131]. In 2023, the number of registered brewery products reached 45,648, showcasing the diversity available in the Brazilian market. Regarding exports, Brazil experienced a significant increase, exporting a total of 231,977,494 L of beer, generating revenue of USD 155,788,372 [131]. This growth underscores the importance and expansion of Brazilian beer production on the global stage.

Beer production requires the use of certain mandatory ingredients: water, malt, and hops. Water, which can make up to 90% of the beverage, must be of potable quality. Malt, a fundamental component, provides essential nutrients for the beer-making process and is critical for developing the characteristic aroma and flavor of the beverage. Alcoholic fermentation occurs from the sugars derived from the transformation of malt during the production process. Additionally, hops, used in small proportions, impart the characteristic bitterness to beer. In addition to the mandatory ingredients, adjuncts—raw materials that provide fermentable sources for the brewing wort—may be added, provided they are legally permitted [127,132].

**Figure 3:**
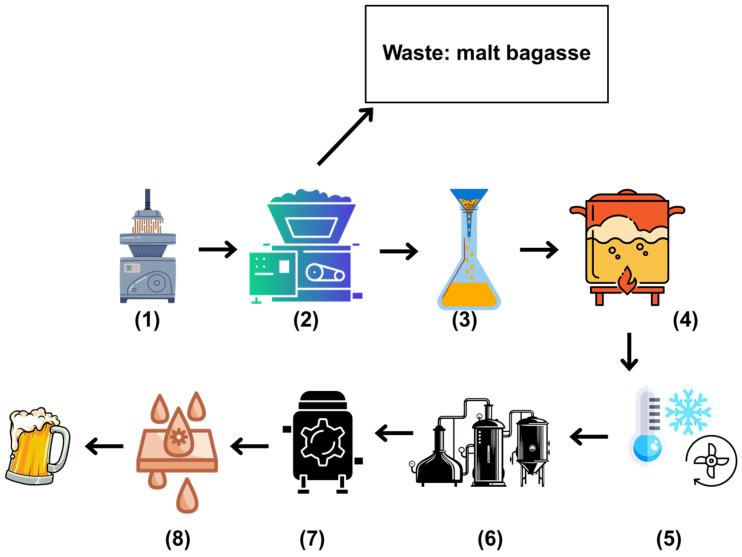
General workflow of the steps involved in beer production.

The production process includes eight essential steps: malt milling (1), mashing (2), wort filtration (3), wort boiling (4), wort treatment (5)—which involves the removal of precipitates, cooling, and aeration—fermentation (6), maturation (7), and clarification (8) [133], as illustrated in Figure 3.

Spent malt grains, a by-product generated during the mashing stage, account for 85% of all residues produced in the brewing process. Despite variations, spent malt grains generally consist of cellulose (16.8–20.6%), hemicellulose (18.4–28.4%), lignin (9.9–27.8%), proteins (15.3–26.6%), extractives (5.2–5.8%), and ash (2.7–4.6%) [134]. To understand the scale of waste generation, it is estimated that every 100 L of beer produced generates approximately 20 kg of spent malt grains, which are commonly used as animal feed [135]. However, due to the high production volumes, spent malt grains are still frequently discarded on land or in landfills [136].

Therefore, exploring alternatives for reusing this residue in other processes is essential. In this context, biotechnology presents a viable and promising solution.

The study conducted by Chimini et al. [136] investigates the use of residues for the production of *Ganoderma lucidum*, a mushroom belonging to the Ganodermataceae family, renowned for its remarkable medicinal properties. This fungus exhibits antiviral, immunomodulatory, anti-inflammatory, antitumor, and antidiabetic effects due to the presence of various bioactive compounds, such as polysaccharides, glycoproteins, alkaloids, steroids, terpenoids, and minerals [137]. In the study, spent malt grains were evaluated as a substrate in different concentrations (0%, 5%, 10%, 15%, and 20%). The production process of *Ganoderma lucidum* involved four main stages: substrate preparation and sterilization, inoculation, incubation, and cultivation. The results showed that the biomass produced ranged from 40.0 to 47.6 g, indicating that the use of spent malt grains is viable up to a concentration of 15%.

The study by Alves et al. [135] examined the application of residues as a substrate for solid-state fermentation in the production of the enzyme tannase. Tannase, an extracellular enzyme produced by fungi, bacteria, and yeasts in the presence of tannic acid, has widespread applications in the food, beverage, and pharmaceutical industries. It is used for producing gallic acid, instant teas, wine color stabilization, and other purposes [138].

Initially, the waste samples were standardized regarding oven drying, pH determination, and particle size, aiming to homogenize the residues for the fermentation process using the fungal strain *Aspergillus niger*. The preparation of the fermentation medium involved adding 20 g of residue (1:1 ratio), salts, and 10% tannic acid. After sterilization, the flasks were inoculated with 2 mL of spore solution and incubated at 32 °C for 120 h. After fermentation, 70 mL of 0.2 M acetate buffer (pH 5.0) were added, followed by shaking at 200 rpm for 1 h. The solution was filtered, centrifuged at 4000 rpm for 15 min, and the enzymatic activity was measured. The enzymatic reaction used a substrate of 0.2% tannic acid in 0.2 M acetate buffer (pH 5.5) with the enzyme extract incubated at 60 °C for 10 min. The reaction was stopped with bovine serum albumin (BSA) and sodium chloride, then centrifuged, and measured at 530 nm. A standard curve was created using commercial tannic acid (0.02–0.14 mg). The best results were obtained using brewery waste and acetate buffer (pH 5.0, 0.2 M) as the extraction agent, with a fermentation medium in a 1:1 ratio, approximately 52% moisture, and incubated for 5 days. The optimal pH for maximum tannase activity was 4.50, with 5% tannic acid as the inducing agent. The enzymatic reaction at 70 °C and pH 4.50 resulted in an increase in enzymatic activity from 0.04 U/mL to 0.19 U/mL by the end of the assays.

Cyanobacteria, such as *Spirulina* (*A. platensis* and *A. maxima*), are photosynthetic microalgae found in both marine and freshwater environments [139]. Considering their commercial relevance, the study by Varandas et al. [140] investigated the cultivation of these species using partial substitution of the synthetic medium with a culture medium derived from barley malt residue (BMR). Initially, the microalgae were cultivated in 250 mL glass flasks with different BMR concentrations (100%, 50%, and 25%), using Zarrouk medium as a control. The cultures were conducted in a climate-controlled chamber (25 ± 1 °C) with fluorescent lighting and a 12 h photoperiod. After identifying the most suitable medium concentration, the cultivation was scaled up to 6 L flasks, and growth was monitored through in vivo fluorescence. Growth parameters, biomass composition, and concentrations of proteins, carbohydrates, lipids, and fatty acids were analyzed. Additionally, the extraction of phycocyanin, polysaccharides, and monosaccharides was performed. Cultures with 50% BMR demonstrated growth comparable to the control. Both *Spirulina* species cultivated in the control medium showed higher chlorophyll content, while phycocyanin was more abundant in *Spirulina platensis*. The highest protein content was observed in *Spirulina platensis* (55.9 g/100 g) and *Spirulina maxima* (53.3 g/100 g) cultivated with BMR. Polyunsaturated fatty acids (PUFAs), such as linoleic and alpha-linolenic acids, had higher percentages in strains grown with BMR, with PUFA content ranging from 57% to 59%. Polysaccharide extraction using hot water and ultrasound revealed higher values in both species cultivated with BMR (8.3% to 11.2%). Exopolysaccharide content was particularly notable in the BMR medium, reaching 191–193 mg/L for both species. Thus, the partial replacement of synthetic medium with BMR proved viable for cultivating these microalgae.

The study by Reis et al. [141] explored a different residue by analyzing the potential of spent brewing yeast as a biosorbent for pharmaceuticals. The yeast, after its use in beer production, was washed, dried, ground, and stored. For the experiments, 0.05 g of yeast was mixed with 100 mL of paracetamol solution (100 μg/mL) in flasks agitated at 125 rpm and 25 °C for 1 h. The samples were filtered, and the paracetamol concentration was measured using a spectrophotometer. Controls without biomass and with biomass in water or buffer were utilized. The amount of paracetamol biosorbed was calculated, and the calibration curve was obtained by diluting paracetamol in distilled water, with absorbance readings at 243 nm. The biosorption conditions were optimized by evaluating the effects of pH (2.0, 3.0, 5.0, 7.0, and 9.0) and temperature (20, 25, 30, and 40 °C) on biosorption at pH 5.0, maintained for 1 h. The effect of contact time was analyzed at 25 °C over 0.5, 1, 3.5, and 15 h. The results showed that in McIlvaine buffer solution at pH 7.0 and 25 °C, the highest adsorption was 136.7 mg/g, while pH 5.0 resulted in the lowest adsorption. Temperatures above 20 °C improved adsorption, but contact times exceeding 3 h were unfavorable due to the desorption of paracetamol from the yeast biomass.

To explore the use of residues in pectinase production, the study conducted by Silva et al. [142] investigated the utilization of these residues as a carbon source for cultivating macrofungi species *Pleurotus djamore* and *Hypsizygus ulmarius* for pectinase production. A central composite rotational design (CCRD) 2^3^ with axial points and three replicates at the central points was employed, totaling 17 different experiments. The design evaluated the effects of three variables: malt bagasse concentration (g/L), temperature (°C), and agitation (rpm). The malt bagasse samples were dried in a ventilated oven at 55 °C for 24 h and then ground into a fine bran. Microbial cultivation was performed in 250 mL Erlenmeyer flasks containing 100 mL of a modified Gern medium, consisting of 5 g/L (NH_4_)_2_SO_4_, 0.2 g/L MgSO_4_, 1 g/L K_2_HPO_4_, 2 g/L yeast extract, 1 g/L casein peptone, malt bagasse bran, and distilled water. The pH was adjusted to 6, and the flasks were sterilized at 121 °C for 20 min. Fungal inoculation was carried out by adding three plugs (9 mm disks containing mycelium) to each flask. The experiments were conducted under specific temperature and agitation conditions for a period of 9 days. The results indicated that for pectinase production by *Pleurotus djamor*, the most influential variables were malt bagasse concentration (30 g/L), temperature (29 °C), and agitation (75 rpm), resulting in an enzymatic activity of 2.544 U/mL. For *Hypsizygus ulmarius*, the key variables were malt bagasse concentration (30 g/L), temperature (24 °C), and agitation (150 rpm), yielding an enzymatic activity of 2.367 U/mL. Both species demonstrated good enzymatic performance at 80 °C.

The utilization of industrial production residues in human nutrition is not only a strategy to reduce waste, but also a crucial step toward promoting more sustainable and healthier diets. The studies highlighted in the table demonstrate a clear trend toward the valorization and use of these residues. Industries must adopt technologies that enable the efficient incorporation of these residues without compromising the quality of final products. Furthermore, it is essential to invest in additional research and development to maximize the potential of these resources.

### Technological Advances Through Intellectual Property Analysis

Table 9 illustrates some patents already filed related to the use of spent malt grains as a by-product of the brewing industry.

The patents filed regarding brewery industry waste reveal a significant trend of innovation and valorization of these by-products. Each patent exemplifies the transformation of waste into a valuable resource, with applications ranging from enzyme production and biotechnological compounds to efficient waste management. The diversity of applications, such as enzyme production, skin care products, manure, and chemical compounds, highlights the importance of researching sustainable practices.

Despite the great potential of whey as a raw material for bioactive compounds, its economic feasibility depends on optimizing hydrolysis and enzymatic conversion processes, which often require sophisticated equipment and stringent parameter control, increasing production costs [148,149,150]. Scaling up to an industrial level is hindered by technical and logistical barriers, especially in regions with limited infrastructure, as well as the need to integrate different technological routes to maximize whey utilization [148,150,151]. Another significant obstacle is the varying regulation of functional foods and bioactive ingredients across countries, which requires investments in safety, efficacy, and labeling studies, delaying market entry [149,150]. Furthermore, the high organic load of whey presents environmental challenges, necessitating sustainable solutions to prevent negative impacts [148,151].

## 6. Biotechnological Applications of Whey: Proteins, Bioactive Peptides, and Lactulose Production

Global milk production has grown significantly over recent decades, driven by increasing demand for dairy products in various regions worldwide. According to the Food and Agriculture Organization of the United Nations (FAO), milk is one of the leading global agricultural products, with production estimated at approximately 906 million tons in 2022. Countries such as India, the United States, China, and Brazil are among the world’s top producers. However, this production growth also presents environmental challenges, particularly in waste management.

The milk production chain generates various solid, liquid, and gaseous residues. Key waste streams include liquid effluents from equipment washing, uneaten animal feed, manure, and residues from dairy product manufacturing. It is estimated that for every liter of milk processed, 3 to 4 L of effluents are generated, making waste management one of the greatest challenges in the dairy industry [152].

Another significant impact of milk production is related to greenhouse gas (GHG) emissions. Dairy farming substantially contributes to methane (CH_4_) emissions due to enteric fermentation in ruminants, along with carbon dioxide (CO_2_) and nitrous oxide (N_2_O), primarily associated with manure decomposition and fertilizer use [153].

Waste management in milk production has been the focus of studies aimed at environmental sustainability, emphasizing the implementation of clean technologies and the optimization of resource use. Biodigesters, for instance, have been identified as an efficient alternative for manure treatment, converting it into biogas and biofertilizers. This approach not only reduces GHG emissions but also promotes circular economy practices in dairy farming [154].

In this context, the adoption of sustainable practices is crucial to minimizing the environmental impacts of milk production and promoting a circular economy in global agriculture. Research and the development of new technologies for waste treatment are essential to ensure that the dairy sector continues to grow sustainably without compromising natural resources or environmental quality.

Whey is a highly nutritious raw material and a rich source of high-biological-value proteins. Proteins found in whey, such as beta-lactoglobulin and alpha-lactalbumin, have diverse biotechnological applications, including the production of bioactive peptides with antioxidant and antihypertensive potential, and the synthesis of lactulose, a disaccharide with prebiotic properties [155].

Whey is a widely produced by-product during cheese manufacturing, serving as an important source of nutrients and bioactive compounds [156]. It contains several high-biological-value proteins, such as beta-lactoglobulin, alpha-lactalbumin, and lactoferrin, which possess functional properties highly valued by the food and pharmaceutical industries [157].

In recent years, biotechnology has explored the potential of these proteins for the production of bioactive peptides, smaller molecules obtained through enzymatic hydrolysis that exhibit various biological activities, including antioxidant, antihypertensive, and antimicrobial effects [158]. Additionally, the production of lactulose, a disaccharide derived from lactose present in whey, has gained prominence for its prebiotic properties and applications in promoting intestinal health [159].

This study aims to review the main biotechnological applications of whey, with a focus on the production of bioactive peptides and lactulose, highlighting their benefits for human health. Table 10 provides a general overview of the chemical composition of concentrated whey, emphasizing its nutritional richness and suitability for various biotechnological applications.

Whey proteins are renowned for their functional and nutritional properties. Among the main proteins are beta-lactoglobulin, which accounts for approximately 50% of the total whey protein content, and alpha-lactalbumin, which makes up around 20% [157]. These proteins are rich in essential amino acids and exhibit high digestibility, making them widely used in the dietary supplement and functional food industries. The hydrolysis of these proteins enables the release of bioactive peptides, which are protein fragments capable of performing specific functions in the body, such as regulating blood pressure and preventing oxidative stress [163].

The production of bioactive peptides from whey proteins has been extensively studied due to their functional potential. These peptides are obtained through enzymatic hydrolysis, a process in which proteolytic enzymes, such as trypsin and chymotrypsin, break proteins into smaller fragments, releasing sequences with specific biological properties [164]. Research indicates that whey-derived bioactive peptides exhibit antioxidant, antihypertensive, and antimicrobial activities. For instance, antioxidant activity has been observed in peptides derived from beta-lactoglobulin, which can neutralize free radicals [165]. Additionally, bioactive peptides have demonstrated the ability to inhibit angiotensin-converting enzyme (ACE), a key mechanism in controlling hypertension [166]. Despite their promising applications, the commercial production of bioactive peptides still faces challenges, such as standardizing hydrolysis processes and characterizing the biological activities of the peptides obtained [167].

Meanwhile, lactulose is a non-digestible disaccharide formed by the isomerization of lactose present in whey. Its primary application is as a therapeutic agent for treating constipation, as well as a prebiotic that promotes the growth of beneficial gut bacteria, such as bifidobacteria [158]. The production of lactulose from whey involves the enzymatic or chemical transformation of lactose, a process widely studied due to the growing demand for natural prebiotics. Lactulose has been incorporated into functional foods and supplements, contributing to gut health and improving the immune system [168].

The use of bioactive peptides and lactulose in food and pharmaceutical products is on the rise. In the food industry, bioactive peptides are incorporated into functional products such as yogurts, protein bars, and beverages due to their antioxidant and antihypertensive properties. In the pharmaceutical sector, lactulose is widely used as a laxative and for the treatment of liver diseases [169]. Additionally, studies suggest that incorporating lactulose into foods can enhance digestion and nutrient absorption, strengthen the immune system, and support the maintenance of gut flora [159].

Biotechnological applications of whey continue to evolve. Research on bioactive peptides paves the way for the development of new nutraceutical products with specific therapeutic properties, such as the prevention of chronic diseases [170]. Meanwhile, lactulose production can be optimized through more efficient enzymatic techniques, contributing to the availability of affordable natural prebiotics for the global market [158].

### Technological Advances Through Intellectual Property Analysis

Intellectual property plays a crucial role in advancing biotechnology, particularly in areas related to bioactive peptides and lactose-derived compounds, driving progress in the nutraceutical and pharmaceutical sectors. For instance, bioactive peptides with therapeutic or functional applications often require extensive research to identify, isolate, and characterize their properties, which patents safeguard to ensure commercial viability. Similarly, innovations in enzymatic processes for the production of compounds like lactulose benefit from patent protection, enabling the commercialization of efficient and sustainable biotechnological solutions.

As may be seen in Table 11, the patents listed highlight diverse applications in the realm of bioactives, focusing on the use of peptides, compositions, and processes, showcasing innovation in peptide-based applications. Meanwhile, other materials describe high-potency sweetener compositions with vitamins, reflecting a focus on multifunctional food additives. Collectively, these patents illustrate the dynamic nature of intellectual property in supporting advancements in biotechnology and functional food development.

Whey is a promising source of proteins, bioactive peptides, and lactulose, with applications across various industries, from food to pharmaceuticals. When hydrolyzed, whey proteins yield bioactive peptides with diverse biological functions, including antioxidant and antihypertensive properties. Lactulose, in turn, stands out as an important prebiotic and therapeutic agent. Advances in biotechnology are expected to further drive these innovations, offering significant potential to enhance health and well-being globally.

## 7. Biotechnological Applications of Coffee By-Products and Waste

The coffee industry plays a crucial role in the global economy, being one of the most traded agricultural commodities worldwide. According to the International Coffee Organization (ICO), global coffee production reached approximately 170 million 60 kg bags during the 2022/2023 harvest, with Brazil, Vietnam, and Colombia standing out as the leading producers [177]. Specifically, Brazil accounts for about 37% of global production, solidifying its position as the largest producer and exporter of coffee [178]. This production chain not only generates employment and drives the economy but also holds strong cultural significance, with coffee being one of the most consumed beverages globally.

However, alongside its economic and social importance, coffee production also generates a significant amount of waste throughout the cultivation and processing stages. It is estimated that only 20% of the total coffee biomass is directly used as a beverage or other derived products, while 80% becomes waste, including husks, pulp, mucilage, and spent grounds [89]. In Brazil, the largest global producer, this waste volume is particularly substantial, reaching millions of tons annually [179].

Coffee is one of the pillars of the Brazilian economy and plays a crucial role in the country’s history and culture. Since its introduction to Brazil, coffee has become one of the main agricultural commodities, with Brazil maintaining its position as the world’s largest producer and exporter of coffee [179]. Coffee production generates millions of direct and indirect jobs, significantly contributing to the national economy by providing income, access to healthcare, and education for many families. Furthermore, coffee is a major source of foreign exchange, with exports accounting for a significant share of Brazil’s total export value [180].

**Figure 4:**
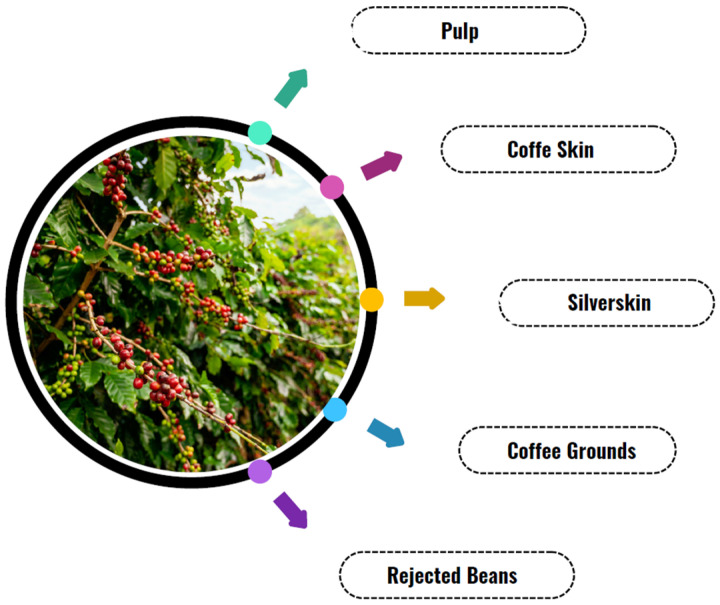
Overview of the main residues generated from coffee processing.

Inadequate management of coffee waste can lead to severe environmental impacts, including soil and water contamination, as well as greenhouse gas emissions resulting from the decomposition of organic matter. However, these residues hold great potential for reuse and have attracted growing interest for sustainable and high-value-added applications [181]. Recent research highlights that coffee waste can be utilized for bioenergy production, extraction of bioactive compounds for the food and pharmaceutical industries, and the creation of sustainable materials such as bioplastics and organic fertilizers [182,183,184]. Figure 4 presents a general overview of the main residues generated in the process chain of coffee.

In this context, the valorization of coffee residues represents a strategic opportunity to promote a circular economy, reduce environmental impacts, and add value to the production chain [185,186]. As shown in Table 12, some important applications of the main by-products of the coffee industry are highlighted.

Coffee residues, particularly spent coffee grounds and husks, are rich in bioactive compounds such as polyphenols, chlorogenic acids, and caffeine, which exhibit antioxidant, antimicrobial, and anti-inflammatory properties [187]. The extraction of these compounds has garnered significant interest from the food industry for the development of functional ingredients and nutritional supplements [194]. Additionally, studies suggest that extracts from these residues can be used as natural preservatives in food products due to their ability to inhibit lipid oxidation and microbial growth [188,195].

The utilization of coffee residues for bioenergy production has gained prominence, particularly in the production of biogas and briquettes [196]. Research indicates that coffee residues can be used to manufacture briquettes, which can replace other fuels in industrial boilers or even for domestic heating in colder regions, utilizing less complex equipment [197]. Anaerobic digestion of coffee husks and mucilage can produce methane, a biofuel suitable for electricity or heat generation [198]. On the other hand, compacting dry residues into briquettes provides a renewable alternative to fossil fuels, offering high calorific value at a low cost.

Coffee residues have also been utilized as agricultural inputs, either as organic compost or bio-stimulants [199,200]. Composting these materials enables the recycling of essential nutrients such as nitrogen, phosphorus, and potassium, which can be used to enrich the soil and enhance plant development [189]. Additionally, studies indicate that aqueous extracts from coffee residues can act as bio-stimulants, promoting plant growth and increasing resistance to biological stresses [188].

Another promising avenue is the use of coffee residues in the cosmetic industry. Substances such as antioxidants and caffeine extracted from spent coffee grounds are widely incorporated into creams, exfoliants, and anti-aging products due to their ability to reduce the effects of free radicals and improve blood circulation [187,188]. This application not only offers a sustainable solution for waste disposal but also adds significant value to this by-product.

Coffee residues are also being explored in the production of sustainable materials. Recent research has demonstrated the use of coffee husks in the manufacture of bioplastics, providing an alternative to conventional petroleum-based plastics [184]. Additionally, spent coffee grounds are being utilized in the development of composites for the construction industry, such as blocks and tiles, due to their good mechanical strength and thermal properties.

Another fundamental aspect is educating and raising awareness among the population and industries about the potential of coffee residues. Campaigns encouraging selective waste collection and domestic reuse, such as using spent coffee grounds for fertilization or homemade cosmetic preparations, contribute to reducing waste and promoting sustainable practices [193].

Despite advances in research, the valorization of coffee residues still faces challenges, such as the economic feasibility of large-scale applications, the standardization of extraction processes, and market acceptance. However, the growing demand for sustainable solutions and the implementation of public policies focused on waste management could drive these initiatives in the coming years. With technological innovation and the promotion of a circular economy, coffee residues have the potential to become a strategic raw material for various industries.

### Technological Advances Through Intellectual Property Analysis

Intellectual property plays a critical role in driving innovation in the coffee sector, particularly in the valorization of its residues for the development of new products. For example, the transformation of coffee waste into bioactive compounds, bioplastics, or functional beverages often involves sophisticated techniques that require intellectual property protection to ensure market competitiveness and promote sustainable innovation. By fostering the development of new uses for coffee residues, patents contribute to a circular economy and encourage the integration of biotechnology in diverse industries, such as presented in Table 13.

The patents listed illustrate the innovative applications of coffee and its by-products, such as a method for manufacturing beverages from waste coffee grounds, fertilizers highlighting the potential for sustainable and functional beverages.

Coffee residues represent a valuable resource with high potential for biotechnological applications. The extraction of bioactive compounds, production of bioenergy, and use as organic fertilizers are just a few of the possibilities for sustainable utilization. With advancements in technology and increased environmental awareness, these solutions are expected to become an integral part of a more efficient and eco-friendly production chain.

## 8. Conclusions

The valorization of agricultural residues, exemplified by coffee and its by-products, underscores the transformative potential of biotechnology in promoting sustainability and innovation across diverse industries. This review highlights the strategic importance of leveraging coffee residues for applications such as bioactive compound extraction, bioenergy production, organic fertilizers, and sustainable materials. By integrating these processes into the production chain, significant environmental and economic benefits can be achieved, aligning with global goals of a circular economy.

Despite the promising applications, challenges such as process standardization, scalability, and market acceptance must be addressed to fully realize the potential of these residues. Intellectual property plays a pivotal role in protecting and promoting innovations in this field, fostering further research and commercial adoption.

The role of biotechnology and microorganisms is central to the development of ingredients and products of industrial relevance. Microbial processes, such as fermentation and enzymatic reactions, are being increasingly harnessed to convert agricultural residues into high-value products, including biofuels, enzymes, and bioactive compounds. These biotechnological processes not only enhance the efficiency and sustainability of production but also open new avenues for the development of novel materials, functional foods, and pharmaceuticals, further driving the growth of a circular economy.

The action of microorganisms, especially bacteria and filamentous fungi, is essential for the reutilization of agro-industrial waste, serving as a technological foundation for sustainable processes. Techniques such as solid-state fermentation, submerged fermentation, and the use of microbial consortia have driven the production of bioactive compounds, such as enzymes, antioxidants, and natural flavors, from agricultural waste, reducing costs and environmental impacts. The inoculation of efficient microorganisms and the adaptation of strains to complex substrates increase the efficiency of degradation and transformation of waste, accelerating composting, improving the quality of the final product, and promoting nutrient release. Moreover, metabolic engineering and the selection of microorganisms with specific capabilities have enhanced the production of secondary metabolites of industrial and environmental interest. The use of waste as a support for microbial immobilization has also shown promise in the bioremediation of pollutants, such as pesticides and heavy metals, making the process more efficient and cost-effective. These advancements signal promising directions for research in environmental and industrial biotechnology, promoting waste valorization and the generation of high-value products.

Looking forward, the synergy between technological advancements, public policy, and industry engagement is essential to scale sustainable solutions. Such as presented, sugarcane, coffee, milk, and cassava residues, alongside other agricultural by-products, have the potential to redefine waste management practices and contribute to a more sustainable, efficient, and resilient bioeconomy.

## Figures and Tables

**Table 1:** Synthesis of valorized agro-industrial residues, applied microorganisms, and involved bioprocesses.

Residue	Products	Microorganisms	Biotechnological Processes
Manioc	Biosurfactants, biogas, bioethanol	*Bacillus subtilis*, *Pseudomonas aeruginosa*, Methanosaeta	Submerged fermentation, anaerobic digestion
Orange	Xylitol, citric acid, XOS, xanthan gum, pectin	*Aspergillus niger*, *Lactobacillus casei*, *Xanthomonas* sp.	Solid and submerged fermentation
Sugarcane	Lactic acid, xylitol, enzymes, bioethanol	*Saccharomyces cerevisiae*, *Aspergillus japonicus*	Fermentation, enzymatic hydrolysis
Whey	Bioactive peptides, lactulose	*Lactobacillus* spp., enzimas proteolíticas	Enzymatic hydrolysis
Brewery waste	Enzymes (tanase, pectinase), fungal biomass, spiruline	*Aspergillus niger*, *Pleurotus* spp., *Spirulina* sp.	Solid-state fermentation, microalgae cultivation
Coffee	Phenolic compounds, biogas, briquettes, bioplastics	Various aerobic and anaerobic microorganisms	Composting, anaerobic digestion

**Table 2:** Composition of cassava processing residues (g/100 g of dry matter) and mineral content (mg/kg of dry matter).

**Residue**	**Crude Protein**	**Crude Fiber**	**Lipids**	**Ash**	**Moisture**
Bagasse	1.12	19.3	2.37	2.84	84.2
Cassava wastewater	2.46	-	-	1.88	96.7
Peels	4.20	29.6	3.26	7.47	82.1
Effluent *	2.92	6.69	1.75	3.16	91.4
**Residue**	**Potassium**	**Calcium**	**Magnesium**	**Iron**	**Sodium**
Bagasse	138	60	129	5.66	93.4
Cassava wastewater	42.7	5.17	22.7	2.21	7.54
Peels	269	122	236	14.7	261
Effluent *	67.7	50.2	33.2	16.2	24

* Effluent: a mixture of whey and fibers. Source: adapted from [32].

**Table 3:** Biosurfactants produced using cassava wastewater as a nutrient source.

Biosurfactant	Substrate	Process Conditions	Microrganism	Operation	Yield (g/L)	Reference
Lipopeptide	Manipueira	30 °C, 150 rpm, 72 h	*Bacillus subtilis* LB5a	Erlenmeyer	3.0	[35]
-	Manipueira	35 °C, 150 rpm, 60 h, aeration 0.63 vvm	*Bacillus subtilis* LB5a	Bioreactor	2.40	[35]
Glycolipid (MEL—B)	Manipueira	0–24 h: 100 rpm, 0.4 vvm; 24–84 h: 150 rpm, 0.9 vvm	*Pseudozyma tsukubaensis*	Bioreactor	1.26	[35]
Lipopeptide	Manipueira	30 °C, 150 rpm, 72 h	*Bacillus* sp. O LB5a	Erlenmeyer	2.0	[37]
Rhamnolipids	Manipueira	25 °C, 100 rpm, 12 dias	*Pseudomonas aeruginosa*	-	0.576	[38]

**Table 4:** Patents related to cassava residues use and valorization.

Year	Patent Number	Applicant(s)	Title	Reference
2008	BRPI0805091	Universidade Federal do Paraná	Process for producing biomass, proteins, and lipids from microalgae using cassava wastewater as a substrate	[49]
2008	CN101423848A	Cofco Corporation	Method for preparing ethanol using raw material containing cassava residues	[50]
2014	CN105624203A	Jiangsu Huating Biotechnology Co., Ltd.	Method for producing biogas from cassava alcohol waste liquid as raw material	[51]
2019	BR102019021055	Universidade Federal do Paraná	Process for producing biohydrogen from agro-industrial effluents and microbial consortium for biohydrogen production	[52]

**Table 5:** Products extracted from orange peel residues.

Products	Technology	Concentration
Pectin	Microwave-assisted extraction	485 mg/mL
Pectin	Conventional acid extraction	381 mg/mL
Flavonoid	Ultrasound technique with extraction solvent (ethanol)	205.2 mg/100 g FW
Carotenoid	Ultrasound-assisted extraction with d-limonene (solvent)	11.25 mg/L

Source: Adapted from Mohsin et al. [73].

**Table 6:** Patents utilizing orange peel residues in technologies.

Year	Patent Number	Applicant(s)	Title	Reference
2013	CN103734519B	Shaoyang University	Method and product for producing an immune-enhancing forage booster using soybean curb residue and orange peel	[78]
2014	CN103783329B	Boon Group Co., Ltd.	Natural forage yolk tonic produced from orange peels and flavedo, and its preparation method	[79]
2015	BR102015018088A2	Universidade Estadual Paulista Julio de Mesquita Filho	Process for obtaining a concentrated lipase extract	[80]
2018	WO2019018914A1	Antonio Luiz Ribeiro de Castro Morschbacker, Patrícia Vargas de Campos, Maurício Oliveira, Alberto Moacir Barbeitos de Freitas	Process for producing ethylene from citrus processing residues, thermoplastic polymers, and plastic articles	[81]
2023	WO2024082045A1	Universidade Estadual de Campinas	Process for producing pectin and xylooligosaccharides from orange juice industrial waste, and xylooligosaccharides	[82]

**Table 7:** General chemical composition of sugarcane bagasse.

Data (%)	[101]	[102]	[103]	[99]
Celullose	50	42.2	37.61	36
Hemicelullose	25	27.6	21.87	-
Lignin	25	21.6	20.6	20
Ashes	-	2.84	-	2.2

**Table 8:** Summary of patents in the utilization and valorization of sugarcane and its by-products.

Year	Patent Number	Applicant(s)	Title	Reference
2009	PI 0903273-8 A2	Mario Clovis Garreffa; Sandro Rogerio de Sousa; Fauze Ali Mere Sobrinho	Biotechnological production of xylitol from organic sugarcane bagasse	[122]
2011	PI 1106897-3 B1	National Institute of Metrology, Standardization and Industrial Quality—INMETRO; Petrobras S.A.	Method for obtaining colloidal suspension for enzymatic tests from plant biomass and its use in detecting enzyme activities on sugarcane bagasse for second-generation ethanol production	[123]
2015	CN104894174A	Jiangnan University	Method for producing succinic acid by taking sugarcane bagasse as raw materials through fermentation	[124]
2018	CN109007545A	Shuzhou Denggao Biotechnology Co., Ltd.	Method for degrading neutral detergent fiber (NDF) and acid detergent fiber (ADF) of sugarcane residues	[125]
2022	BR 10 2022 022208 8 A2	Universidade Estadual de Londrina	Production of citric acid by Aspergillus welwitschiae strains from sugarcane residues	[126]

**Table 9:** Patents utilizing brewery waste in technological processes.

Year	Patent	Applicant	Title	Reference
2011	BRPI1101711B1	Universidade Estadual de Campinas	Process for the production of ethyl hexanoate via biotechnology using synthetic medium and agro-industrial residues and its use	[143]
2013	BR102013031848A2	Universidade Federal do Paraná	Production of microbial dextranase by solid-state fermentation using malt bagasse as substrate and/or support	[144]
2018	WO2018136234A1	Ian Mackay Carlos Greden	Process for the production of protein concentrate or isolate and thermochemical cellulose raw material from brewery residual alcohol	[145]
2016	CN106588487A	Dalian Sem Biological ENG Tech Co., Ltd.	Method for preparing bio-bacterial manure by means of brewery waste diatomite	[146]
2023	CN117838561A	Guilin Kangsheng Tech Co., Ltd.	Method for preparing skin care product from waste beer yeast paste or waste beer yeast liquid, dry rice maltose residues	[147]

**Table 10:** Chemical composition of concentrated whey.

Data (%)	[160]	[161]	[162]
Protein	34.2	38	37
Lipids	0.2	1	0.7
Lactose	54.8	54	51
Ashes	4.7	4	6.6

**Table 11:** Patents aiming for the valorization of whey in technological processes.

Year	Patent Number	Applicant(s)	Title	Reference
2010	BR112012014660-2B1	Stokely-Van Camp, Inc.	Isotonic and rehydration drinks and method for their production	[171]
2008	US8383183B2	The Coca-Cola Company, Atlanta, GA, USA	Sweetness enhancers, sweetness enhanced sweetener compositions, methods for their formulation, and uses	[172]
2006	US9173425B2	The Coca-Cola Company, Atlanta, GA, USA	High-potency sweetener composition with vitamin and compositions sweetened therewith	[173]
2017	BR 112019000731-8 B1	Arla Foods Amba	Method for preparing a gelatinizable whey protein composition by acid and method for preparing a food product	[174]
2022	BR 102022018062-8 A2	Fundação Vale do Taquari de Educação e Desenvolvimento Social FUVATES	Use of peptide, composition, and process for the preparation of a composition	[175]
2023	CN117683074A	Shandong Lvbang Biotechnology Co., Ltd.	*Bacillus* cereus, protease inhibitor and method for increasing yield of protein residue	[176]

**Table 12:** Applications of major by-products of the coffee industry.

By-Products	Applications	References
Coffee husk	Enzyme production; phenolic compounds	[187,188]
Coffee pulp	Production of bioethanol; production of volatile aromatic compounds; natural flavorings; animal feed	[189]
Silverskin	Insulin secretion enhancers; bread production	[190,191]
Coffee ground	Soil fertilizers; cationic adsorbents in wastewater treatment; alcoholic beverage production; liquid Fuel production from ground coffee oil	[192,193]

**Table 13:** Patents utilizing coffee by-products in biotechnological processes.

Year	Patent Number	Applicant(s)	Title	Reference
2015	KR101801655B1	H & K Global; Jae Woong Jung; Jun Ik Son	Composting method of soil conditioner and food waste comprising spent coffee ground fermented by microorganisms and fermented compost made by the same	[201]
2022	CN114868856A	University of Jiangsu	Method for preparing fermented beverage by taking waste coffee grounds as raw materials	[202]
2024	CN118206403A	Yunnan Bolong Biotechnology Dev Co., Ltd.	Method for producing organic fertilizer by using coffee waste	[203]

## Data Availability

No new data were created or analyzed in this study. Data sharing is not applicable to this article.
